# Is the Concept of Pseudoneurotic Schizophrenia Still Valid? A Case Series and Revisiting the Topic

**DOI:** 10.7759/cureus.4227

**Published:** 2019-03-11

**Authors:** Navjot Brainch, Sanya Virani, Penchaya Atiwannapat, Souparno Mitra, Yassir Mahgoub

**Affiliations:** 1 Psychiatry, Maimonides Medical Center, Brooklyn, USA; 2 Psychiatry, Army Medical Corps, Edison, USA

**Keywords:** pseudoneurotic schizophrenia, borderline states, psychosis

## Abstract

We describe two cases to emphasize the importance of recognizing symptoms of psychosis underlying a constellation of neurotic symptoms, and to highlight the overlap and potential for misdiagnosis with personality and anxiety disorders. We also provide an overview of pseudoneurotic schizophrenia and familiarize readers about the challenges in making accurate diagnoses in light of this term used in the past. We refer to cases in the literature and point out the implications of this concept on diagnosis, management, and prognosis. Based on the management strategies deployed for the two cases described, we finally recommend that it is imperative to perform accurate and detailed assessments and take into consideration the evolution of the concept of pseudoneurotic schizophrenia to currently accepted DSM-V disorders, in order to effectively treat patients.

## Introduction

Pseudoneurotic schizophrenia was the term coined by Hoch and Polatin in the 1940s to describe patients who presented with “neurotic” facade, which concealed thought, emotional and behavioral impairment of regulation, integration, and stemmed from “psychotic” process [1]. Due to broad and vague diagnostic criteria that bordered between psychosis and neurosis, the term became synonymous with “borderline states”, creating clinical confusion.

We present two cases, initially deemed to exhibit predominant personality and anxiety disorder features, but later revealed underlying psychosis with significant formal thought disorder on detailed assessment, and revisit the concept.

## Case presentation

Case 1

Mr. X, a 29-year-old male with past psychiatric diagnoses of major depressive disorder, generalized anxiety disorder, borderline personality disorder, and history of multiple suicidal attempts by overdose in context of feeling “void”, “numb”, “bored” and “overwhelmed”. He presented with vague and unclear anxiety, panic attacks, feelings of generalized discomfort, and reports of passive suicidal ideation. Upon evaluation, he was guarded, oddly related, occasionally distracted, and unable to provide relevant or logical answers to most questions due to prominent blocking and disorganization of thoughts. He exhibited ambivalence in basic decision-making processes related to treatment and disposition and reported confusion about his sexual orientation and preference. Throughout his stay in the hospital, he displayed poor interpersonal and coping skills, impaired impulse control, and a gradual decline in functioning that had commenced 11 years prior to the current presentation. Records from previous outpatient visits and inpatient hospitalizations described presentations of numbness, emptiness, poor impulse control and interpersonal skills as well as superficial expression of emotions which led to diagnoses of borderline personality disorder, generalized anxiety disorder, and major depressive disorder. Notably, he had also failed several antidepressant trials. He was prescribed an oral dose of 50 mg of quetiapine which was titrated upwards to 300 mg, at which point there was significant improvement in symptoms of anxiety, depression, and suicidal ideation. His thought process became somewhat linear; he slowly started gaining insight into his condition and was able to communicate. 

Case 2

Ms. Y, a 39-year-old female with a history of major depression and anxiety, diagnosed and managed by her therapist for a few months prior to her hospitalization, was referred to the emergency room when she decompensated, reporting vague symptoms of paranoia, auditory and visual hallucinations. She was noted to be anxious, apprehensive, and perplexed, demanding to be seen repeatedly for reassurance. Vagueness, tangentiality, and circumlocution pervaded her conversations. She narrated events that permitted the team to learn about her poor boundaries with strangers, inability to maintain healthy relationships, and difficulty with keeping steady jobs. Borderline personality traits were initially suspected but collaterals from her aunt described her as an “odd, paranoid person” with poor interpersonal skills and said that she had often seen her talking to herself. However, being distant and not causing nuisance in the community, she had not drawn attention to herself and had never been hospitalized. A diagnosis of schizophrenia was formulated and her symptoms of anxiety, hallucination, and thought disorganization improved on oral risperidone gradually titrated to a daily dose of 6 mg.

## Discussion

Hoch and Polatin coined the term “pseudoneurotic schizophrenia” in 1940s to describe a group of patients who presented with symptoms of intense anxiety and mood disorder which occurred in the context of underlying psychosis and proposed diagnostic criteria 10 years later [1]. The proposed diagnostic criteria for pseudoneurotic schizophrenia consisted of primary clinical symptoms (disorders of thinking and association, disorders of emotional regulation, and disorders of sensorimotor and autonomic functioning) together with secondary clinical symptoms (pan-anxiety, pan-neurosis, and pan-sexuality) [2]. The use of term became obsolete after the diagnosis of borderline personality disorder was included in the DSM-III in 1980 [3].

These diagnostic criteria faced criticism due to its broadness, vagueness, and questionable accuracy. Hoch et al. pointed out that, “The organization of the outline (the proposed diagnostic criteria) has many deficiencies. However, they believed that it can lead to more definite and more refined clinical observations, while doing much to maintain a holistic concept of the patient” [2]. Because of the increased recognition of borderline personality disorder as a separate diagnosis, not primarily psychotic in nature, the use of pseudoneurotic as well as other seemingly confusing terminologies such as incipient or latent schizophrenia became less common.

Though no longer acknowledged as a clinical entity, the concept still holds significance in current psychiatric practice because of potential implications of the diagnosis and management. A five to 20-year-follow-up study of 109 patients with pseudoneurotic schizophrenia was published by Hoch et al., which reported a significant incidence of subsequent hospitalization following initial contact [4]. It was also noted that 20% of these patients developed overt schizophrenic symptoms and 10% attempted suicide [4]. 

In our first case, our patient presented with symptoms of depression, distressing anxiety, and emotional dysregulation for years with no overt signs or symptoms of overt psychosis. His poor social interactions, unstable relationships, and emotional outbursts made all previous providers suspect borderline personality disorder. However, on detailed assessment and more collateral information, it became evident that he was notably indecisive, engaged only superficially in conversations and was unable to provide details. Additionally, he continued to experience bouts of sudden unexplained sadness, depression, and anger that are likely to have stemmed from a primary psychotic thought disorder. And although the classical symptoms of auditory or visual hallucinations and paranoia were the not cornerstones of his presentation, the patient remained oddly related, unable to connect on social levels, and continued to show significant functional decline, which might bear some semblance to negative symptoms. He was treated with antidepressants for years which aimed to target his low mood, suicidal thoughts, and anxiety but responded poorly and had prolonged hospital stays. There was a clear thought block that was noticed while interviewing the patient. He was able to carry a conversation with staff but with attempts to clarify and get more details about his symptoms, there was noticeable thought block, confusion, oddity and inability to relate with the team. Before he was started on quetiapine, he stayed in his room all day and was not interested in interaction. Few days after we started quetiapine, thought block resolved, he was able to relate better with the team and with other peers in the unit, his ambivalence and suicidal thoughts improved. In the case of borderline personality disorder, mood stabilizers and antipsychotics can help in controlling the mood swings, anger outburst and suicidality, which was not the symptoms addressed in this case. The improvement he showed with 300 mg of quetiapine is testament to the fact that some underlying psychotic symptoms were not being adequately targeted.

Psychosis was somewhat more evident in the second case. This patient presented with vaguely reported paranoia, auditory, and visual hallucinations and her family confirmed it. She had poor interpersonal, social, and functional abilities for many years. She kept to herself and had very limited social interaction. She did cut ties with many people and family members but as she was not problematic, no one pushed for evaluation or treatment. Borderline personality disorder traits were initially suspected as she appeared dramatic, shallow, and demanding and much like the above case, she also showed marked improvement with oral risperidone. In summary, both patients met the Hoch and Polatin definition of pseudoneurotic schizophrenia on initial assessments and had failed multiple antidepressants trials (that paradoxically made some of symptoms worse), but they eventually responded well to antipsychotics. 

The challenges in teasing apart primary psychotic symptoms in such presentations and making accurate diagnoses are manifold. Distracting symptoms of anxiety, lack of clarity of presenting complaints and unclear previous psychiatric history are few of them. Moreover, providers often view patients with the diagnosis of borderline personality disorder with some bias, which only tends to compound the issue [5-6].

Limited information is available about the treatment of pseudoneurotic schizophrenia when such a diagnosis was made in the past. In the 1962 Hoch et al. follow up study, it was pointed out that, “Since the major part of our study was concluded before the newer psychopharmacological agents were available, their use does not figure in this report” [4]. There is paucity of data on the use of antipsychotics in such patients. In a case report published by Connor et al. it was reported that “his (the patient’s) low mood responded to antidepressants but his anxiety symptoms, impaired concentration and thought disorder worsened and his somatic concerns reached delusional intensity. The addition of olanzapine resulted in a dramatic improvement in all symptoms, including his anxiety.” The poor response to antidepressants, but significant response to antipsychotics is very similar to the cases we presented.

Hafner et al. asserted that behavioral and symptom profiles in depressive and psychotic prodromes are difficult to tell apart [7]. In recent retrospective studies, it has been noted that the presence of depression and anxiety along with the psychosis prodrome predicts the possibility of future onset of psychosis with as much as 30%-40% transitioning to the first episode of psychosis within 12 months [8-10]. We may argue that the two discussed cases met the criteria for pseudoneurotic schizophrenia upon initial encounters [2]. However, with detailed assessment actually met criteria for schizophrenia DSM-V [11].

## Conclusions

The previously made criteria were wide, vague, and commonly shared by personality disorder, and hence, it was abandoned for decades. This unique and interesting presentation deserves further attention and more research to identify targeted strategies for better management of these patients.
